# Acute mesenteric ischemia with abdominal skin mottling

**DOI:** 10.1002/jgf2.737

**Published:** 2024-10-06

**Authors:** Masahiro Yabe, Yuji Nomoto, Hitomi Watanabe

**Affiliations:** ^1^ Department of General Internal Medicine Niigata City General Hospital Niigata Niigata Japan; ^2^ Department of Palliative Care Medicine Niigata City General Hospital Niigata Niigata Japan; ^3^ Department of Radiology Niigata City General Hospital Niigata Niigata Japan

**Keywords:** abdominal skin mottling, acute mesenteric ischemia, circulatory failure, skin mottling

## Abstract

We report a rare case of a 90‐year‐old woman with Stage IV lung cancer awaiting transfer to hospice care who developed sudden abdominal and knee skin mottling. Elevated inflammatory markers on blood tests and emergent computed tomography led to a diagnosis of acute mesenteric ischemia, and the patient passed away 7 h later. Skin mottling indicates decreased blood flow in the gastrointestinal tract and is observed during mesenteric ischemia. Abdominal skin mottling may serve as an important physical finding that is detected early and suggests circulatory failure and intestinal ischemia.

## Graphical abstract

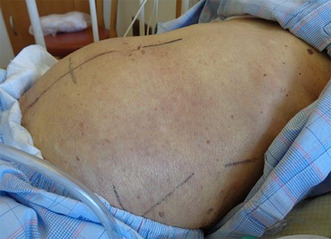

## 1 BACKGROUND

Acute mesenteric ischemia is a potentially fatal condition, necessitating an early diagnosis in any clinical setting.^1^ It should be considered when severe abdominal pain is disproportionate to the findings of a physical examination.^1^ Diffuse abdominal tenderness, symptoms of peritoneal irritation, and abdominal distention indicate mesenteric ischemia; however, there are no physical findings with diagnostic utility.^2^ In this report, we describe a unique case of acute mesenteric ischemia that presented with skin mottling not only around the knee but also in the abdomen. Abdominal skin mottling is a physical finding detected early and suggests acute mesenteric ischemia.

## 2 CASE PRESENTAION

**FIGURE 1:**
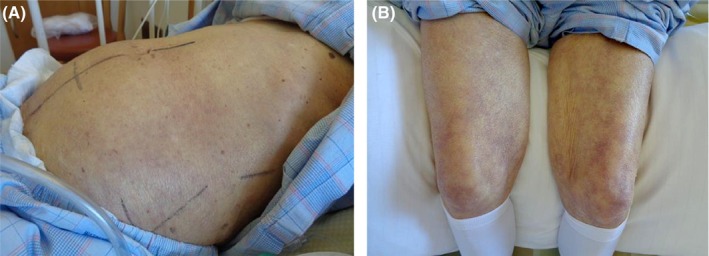
(A) The patient's abdomen appears distended and taut, with skin mottling throughout. (B) Skin mottling is evident over the patient's knees.

A 90‐year‐old woman presented with sudden abdominal distention, dark‐brown emesis, and decreased urine output during her hospital stay. She was hospitalized for a stroke and was awaiting transfer to hospice care because he had Stage IV advanced lung cancer, and her prognosis was estimated for a few months. Before the onset of symptoms, her systolic blood pressure was stable at around 110 mmHg, and urine output was maintained at 1000–1500 mL/day until the previous day. Her vital signs were as follows: blood pressure, 155/88 mmHg; pulse, 91 bpm; temperature, 36.4°C; respiration rate, 27 breaths/min; and oxygen saturation, 94% on room air. Her Glasgow Coma Scale score was 15 (E4, V5, M6). Physical examination revealed a distended, tense abdomen with no tenderness. Skin mottling was observed throughout the abdomen (Figure 1A) and extended to the legs (Figure 1B). There was a cold sensation in the peripheral extremities but there were no findings suggestive of cholesterol embolism or vasculitis.

**FIGURE 2:**
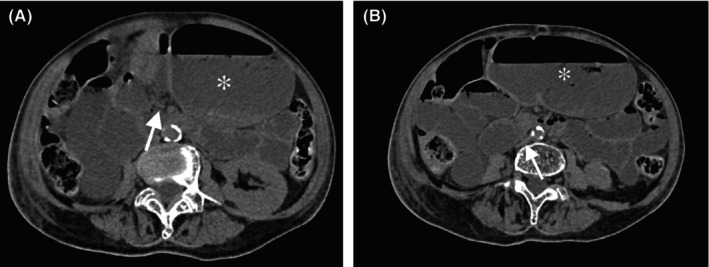
(A) Emergent plain abdominal computed tomography reveals paralytic ileus of the stomach (*) and small intestine, as well as narrowing of the superior mesenteric vein (arrow). (B) Emergent plain abdominal computed tomography reveals paralytic ileus of the stomach (*) and small intestine, as well as flattening of the inferior vena cava (arrow).

Laboratory findings revealed the following: C‐reactive protein, 354 mg/L (reference value: <1 mg/L); white blood cell count, 10.3 × 103 cells/μL; neutrophils, 89.2%; eosinophils, 0.3%; aspartate transaminase, 140 U/L; alanine transaminase, 70 U/L; lactase dehydrogenase, 371 U/L; and serum creatinine, 0.73 mg/dL. Emergency plain computed tomography revealed paralytic ileus of the stomach and small intestine, smaller superior mesenteric vein sign (narrowing of the superior mesenteric vein) (Figure 2A), flattening of the inferior vena cava (Figure 2B), and collapse of the renal and hepatic veins. However, portal venous gas, intestinal emphysema, or mesenteric edema was not observed.

Due to its sudden onset, acute mesenteric ischemia with circulatory failure was diagnosed. Palliative care was provided based on the underlying disease, and the patient passed away 7 h later.

## 3 DISCUSSION

We report a rare case of a patient who showed abdominal and knee skin mottling with acute mesenteric ischemia. Skin mottling is a prevalent clinical sign in patients with circulatory failure, including those with cardiogenic shock and septic shock.^3^, ^4^ Vasoconstriction of small blood vessels due to circulatory failure leads to aberrant blood flow in the skin. The area around the knee joint is a site where reduced blood flow can be easily identified and mottling severity can be scored.^4^ A study involving 131 patients with mesenteric infarction who underwent surgery reported that skin mottling was observed in 45.8% of patients, of whom 60.7% died within 72 h and 32.4% survived.^5^ Thus, skin mottling is commonly observed in acute mesenteric ischemia but our case was unique in that skin mottling was observed around the knee and in the abdomen.

Acute mesenteric ischemia causes injury to the gastrointestinal mucosa and submucosa as the oxygen supply deteriorates and prolongs. This leads to bacterial translocation and activation of the inflammatory response, further exacerbating vasospasm and resulting in the progression of intestinal ischemia.^1^ The skin, muscles, and gastrointestinal tract are peripheral vascular beds susceptible to hypoperfusion, causing a compensatory reduction in skin perfusion in response to tissue hypoperfusion.^6^ Brunauer^7^ reported a correlation between the Mottling score and the kidneys' pulsatility index, suggesting that mottling skin reflects decreased renal blood flow and hypoperfusion.^7^ In our case, a sudden onset decrease in urine output was observed, suggesting that acute intestinal ischemia caused a rudecrease in renal blood flow due to renal vasoconstriction. Thus, both acute mesenteric ischemia and the resulting reduction in renal blood flow may have caused greater hypoperfusion of skin tissue in combination, leading to mottling skin not only around the knee but also in the abdomen.

Although reports of abdominal and generalized skin mottling associated with mesenteric ischemic disease are rare in the current literature, Yazigi et al.^8^ reported two patients who died from acute mesenteric infarction and abdominal and generalized skin mottling. Tominaga et al.^9^ reported a case in which abdominal mottling appeared concomitantly with acute gastric dilatation and resolved with the release of gastric dilatation. Gastric dilatation may compress the inferior epigastric artery, causing transient peripheral circulation insufficiency of the lower abdomen. Furthermore, abdominal mottling has been observed in patients with fatal sepsis.^10^ In our patient, abdominal skin mottling was observed shortly after disease onset, coinciding with the occurrence of acute mesenteric ischemia and ultimately leading to death. Therefore, patients presenting with abdominal skin mottling may be critically ill with intestinal ischemia and should be managed with caution.

## 4 CONCLUSION

We presented a rare case of acute mesenteric ischemia in an older adult patient who exhibited abdominal skin mottling as a clinical sign. Abdominal skin mottling can be recognized early and is considered a physical finding indicative of acute mesenteric ischemia.

## CONFLICT OF INTEREST STATEMENT

The authors have stated explicitly that there are no conflicts of interest in connection with this article.

## ETHICS STATEMENT

Ethics approval statement: Not applicable.

Patient consent statement: The patient's son provided written consent authorizing publication.

Clinical trial registration: None.
